# The Development of a European Registry for Facial Dysostosis Syndromes: A Delphi-Guided Approach

**DOI:** 10.1097/SCS.0000000000011695

**Published:** 2025-07-23

**Authors:** Victor L. van Roey, Saranda Ombashi, Irene M.J. Mathijssen, Åsa A. Munkhammar, Pamela M. Åsten, Anouar Bouzariouh, Charlotte Célérier, Caroline Dana, Marieke van Dooren, Mariët Faasse, Brigitte Fauroux, Peter Frykholm, Arnoud Heinen, Arja Heliövaara, Koen F.M. Joosten, Marizela Kljajic, Eva Larsson, Sjoukje Loudon, Ivana Marinac, Montserrat Munill Ferrer, Henriette Poldermans, Eva-Lena Stattin, Marloes Streppel, Malin Svensson, Briac Thierry, Stephen T.H. Tjoa, Alexandra Topa, Elin Weissbach, Eppo B. Wolvius, Víctor Zafra Vallejo, Sarah L. Versnel

**Affiliations:** * European Reference Network for Rare and/or Complex Craniofacial Anomalies and Ear, Nose, and Throat Disorders, Rotterdam, The Netherlands; † Erasmus University Medical Centre Rotterdam, Rotterdam, The Netherlands; ‡ Uppsala University Hospital, Uppsala, Sweden; § Oslo University Hospital, Oslo, Norway; ∥ Hôpital Necker – Enfants Malades, Assistance Publique – Hôpitaux de Paris, Paris, France; ¶ Dutch Patient and Parent Society for Congenital Craniofacial Conditions (LAPOSA), The Netherlands; # Helsinki University Hospital, Helsinki, Finland; ** Sahlgrenska University Hospital, Institute of Biomedicine, Sahlgrenska Academy, University of Gothenburg, Gothenburg, Sweden; †† Rare Diseases Croatia, Croatia; ‡‡ Hospital Vall d’Hebron, Barcelona, Spain; §§ University Hospital 12 de Octubre, Madrid, Spain

**Keywords:** Acrofacial dysostosis, Delphi technique, mandibulofacial dysostosis, Miller syndrome, Nager syndrome, Treacher Collins syndrome

## Abstract

Facial dysostosis syndromes (FDS) are rare congenital conditions that significantly impact facial function and appearance. At the time of this writing, standardised monitoring protocols for FDS are lacking, hampering research, and evidence-based care. Thus, a comprehensive dataset was developed within the European Reference Network for Rare and Complex Craniofacial Anomalies (ERN CRANIO). Candidate data elements were identified through a systematic literature review (1985–2024) and supplemented with elements from existing ERN CRANIO datasets and expert panel suggestions. A Delphi survey was then conducted among 61 clinicians and 3 patient representatives to assess each element’s relevance and reliability using a 9-point Likert scale. A subsequent hybrid consensus meeting with the expert panel shaped the final dataset, ensuring comprehensive coverage, avoiding overlap, and determining the appropriate timing for data collection. Of 200 data elements that entered the Delphi voting, 98 were strongly recommended, 102 scored neutral, and none were strongly discouraged. Ultimately, 110 elements were included, organised into 2 levels: Level 1, comprising exclusively patient-reported and parent-reported outcome measures; and Level 2, encompassing patient characteristics, treatment information, clinical outcomes, and imaging/diagnostics. This newly developed dataset marks the first international registry for FDS, offering considerable potential for collaborative research, cross-centre comparisons, and substantial improvements in care for patients with FDS worldwide. Real-world implementation will be essential to evaluate its feasibility and guide further refinements.

Facial dysostosis syndromes (FDS), such as Treacher Collins, Nager, and Miller syndromes, are rare congenital conditions that primarily affect the development of the face.^1–4^ Patients with FDS often experience a wide range of functional impairments, including difficulties with breathing, feeding, speech, hearing, and vision, which significantly impact their quality of life.^5,6^ In addition to craniofacial anomalies, some of these syndromes also come with extracranial anomalies, particularly affecting the limbs.^7^

The rarity and phenotypic variability of these syndromes have posed significant challenges in establishing standardized guidelines for diagnosis and treatment.^8^ As a result, treatment approaches have traditionally relied on expert opinion and institutional preferences. To address the lack of guidance, a clinical consensus statement for FDS was developed in 2024 within the European Reference Network for Rare and Complex Craniofacial Anomalies (ERN CRANIO).^9^ This consensus statement provides a first framework for diagnostic and treatment practices, offering guidance to improve and standardize care across centers.

However, despite the consensus statement, scientific evidence on the optimal diagnostic and treatment practices for FDS remains scarce.^10^ Further research and international collaboration are essential to gather more robust data for these rare conditions. At the time of this writing, such collaborations are hindered by variations in monitoring protocols, with centers collecting different outcome measures at varying time points.

To overcome these challenges, the development of a European registry for FDS was initiated to standardize data collection across multiple centers, enabling better comparison of treatment outcomes and improvement of patient care. The aim of this article is to describe the process of developing an internationally applicable and comprehensive dataset that can be used to monitor, evaluate, and compare the quality of care for FDS patients across Europe.

## METHODS

The FDS dataset was developed through a systematic process, starting with a systematic literature review to identify candidate data elements. These elements were complemented with data elements from other ERN CRANIO datasets (ie, cleft lip and palate, craniofacial microsomia), as well as data elements suggested by members of the expert panel based on their clinical experience and expertise. The expert panel consisted of health care providers from various specialties involved in the treatment of FDS patients, representing 12 ERN CRANIO centers across 8 European countries (Supplemental Digital Table 1, Supplemental Digital Content 1, http://links.lww.com/SCS/I122). The panel also included 3 patient representatives affiliated with national patient organizations for rare congenital craniofacial conditions (ie, Rare Diseases Croatia and Laposa).

After gathering the initial data elements, a Delphi survey was conducted to reach consensus on the reliability and relevance of these elements for monitoring, evaluating, and comparing the quality of care for FDS in Europe. Some data elements were not subject to the Delphi process. These “common data elements” were mandatory for inclusion to comply with the European Commission standards for rare disease registries. These elements include essential information such as patient demographics, diagnosis, disease history, care pathways, and research-related details. ERN CRANIO expanded this list with additional data elements specific to craniofacial conditions.

Given the multidisciplinarity and complexity of FDS and their treatment, as well as the broader requirements of an international registry, the results of the Delphi survey could not be used as the sole basis. Instead, the results served as the primary guidance for further dataset development. Thus, a hybrid (ie, online and in-person) consensus meeting with the expert panel was held to ensure that all critical aspects of FDS were represented in the dataset, to avoid overlap between elements, and to determine the appropriate timing of data collection for each element. Finally, the resulting dataset concept was circulated to the expert panel for confirmation and final remarks.

### Literature Review

An up-to-date literature review was conducted to identify candidate data elements for inclusion in the FDS dataset, using the same search strings as in the clinical consensus statement.^9^ Searches were performed in Embase, MEDLINE/PubMed, Cochrane, Web of Science, and CINAHL, targeting studies published between January 1985 and May 2024 (Search strings in Supplemental Digital File 1, Supplemental Digital Content 2, http://links.lww.com/SCS/I123). Studies were included if they reported on diagnostic or treatment outcomes in FDS patients, with a minimum sample size of 5. Studies focusing solely on etiology or pathogenesis were excluded, as well as conference proceedings, commentaries, letters, narrative reviews, editorials, dissertations, and unpublished work.

The identified data elements were grouped into categories: “patient-reported outcome measures (PROMs) and questionnaires,” “patient characteristics,” “clinical outcome measures,” “imaging and diagnostics,” and “treatment information” (Supplemental Digital Table 2, Supplemental Digital Content 1, http://links.lww.com/SCS/I122). Elements found to be unreliable or strongly discouraged in the literature were excluded from further consideration before the Delphi process. For the imaging and diagnostics category, direct storage of files in the registry was not feasible due to concerns about maintaining patient anonymity (eg, facial photography) and file size limitations (eg, CT scans). Therefore, most data elements in this category instead register the availability of imaging and diagnostic data are available for each patient without storing the actual files.

### Delphi Process

A single round of Delphi voting was conducted to reach consensus on the reliability and relevance of the identified data elements. Health care providers from 12 ERN CRANIO centers with expertise in FDS and 3 patient representatives were invited to vote in the Delphi survey. Experts were urged to vote only on data elements related to their area of expertise. Thus, each data element was rated on a 9-point Likert scale, where 1 indicated “complete disagreement,” and 9 indicated “complete agreement.” Data elements were highly recommended for inclusion in the dataset if they received a median score of ≥8, with at least 75% of responses in the highest tertile (scores of 7, 8, or 9).^11^ Conversely, if data elements received a median of ≤2, with at least 75% of responses in the lowest tertile (scores of 1, 2, or 3), their inclusion was highly discouraged. The inclusion of the remaining “neutral” elements was also discouraged, but these elements were still up for debate in the expert panel to ensure that all critical aspects were covered in the dataset.

### Consensus Meeting

Following the Delphi process, a hybrid consensus meeting was held in November 2024 during the ERN CRANIO annual meeting in Gdansk, Poland. During this meeting, the expert panel reviewed the set of elements that were highly recommended for inclusion to avoid overlap between elements (eg, degree of hearing loss versus pure tone average). Subsequently, the neutral data elements were also discussed for inclusion to ensure that all critical aspects of FDS were represented. Adjustments were made to these elements if considered necessary by the expert panel.

In addition, the expert panel determined the appropriate timing for data collection of each element by placing them on a common timeline used across multiple ERN CRANIO datasets. This common timeline was chosen to facilitate the registration of patients who fall under multiple datasets, such as those with both a facial dysostosis syndrome and a cleft lip and palate. Guided by the recommendations of Ombashi et al,^12^ it included the following age categories: initial presentation, 0 to 2 years, 3 to 4 years, 5 to 7 years, 8 to 9 years, 10 to 13 years, 14 to 16 years, and 17+ years. Due to the individualized nature of treatment for FDS, elements from the categories “treatment information” and “imaging and diagnostics” were left independent of this timeline to allow flexibility in capturing these details as they occur.

### Dataset Categorization

After the hybrid consensus meeting, the final dataset was categorized into 2 levels to enhance accessibility and participation across centers, acknowledging the limited financial and time resources in some centers and/or countries. A “level 1” dataset was defined to exclusively include PROMs or questionnaires that imposed no additional burden on clinicians, as these elements were completed by patients or their representatives. In contrast, the “level 2” dataset comprised the remaining data elements, including patient characteristics, treatment information, clinical outcome measures, and imaging and diagnostics. It should be noted that the data elements in both levels were intended to reflect standard care or best care practices for patients with FDS. The finalized dataset was then circulated to the expert panel for confirmation and final remarks, concluding the dataset development.

## RESULTS

**FIGURE 1:**
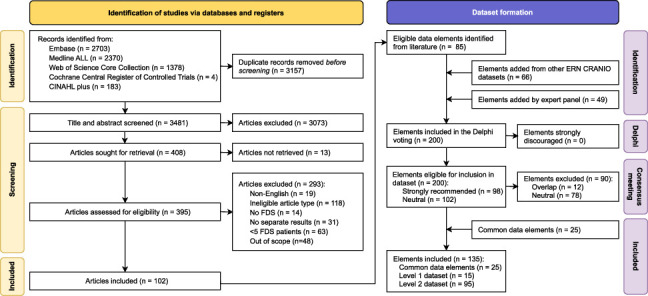
Flow chart of the article selection and dataset formation process.

Figure 1 illustrates the process of article selection, Delphi voting, and dataset formation. After deduplication and screening of 3481 articles, 102 met the eligibility criteria. From these, 85 eligible data elements were identified, complemented by 66 data elements from other ERN CRANIO datasets, and 49 additional elements proposed by expert panel members.

In total, 200 data elements were subjected to Delphi voting. Of these, 98 (49.0%) were strongly recommended for inclusion, none were strongly discouraged, and 102 (51.0%) scored neutrally. The voting process involved 61 experts from various specialties and 3 patient representatives (Supplemental Digital Table 1, Supplemental Digital Content 1, http://links.lww.com/SCS/I122).

Through the consensus meeting with the expert panel, 12 strongly recommended data elements were excluded due to overlap with other elements (Supplemental Digital File 2, Supplemental Digital Content 3, http://links.lww.com/SCS/I124). In addition, 24 neutral-scoring elements were included to ensure comprehensive coverage. Ultimately, 110 data elements were included in the FDS dataset with 15 categorized in the level 1 dataset and 95 in the level 2 dataset, besides the 25 common data elements. A complete list of all included data elements, their response options, timing, and Delphi scores is provided in Supplemental Digital File 2 (Supplemental Digital Content 3, http://links.lww.com/SCS/I124).

### Common Data Elements

The dataset includes 25 common data elements. These include demographic information such as date of birth, sex, country of residence, and patient status (eg, alive, lost to follow-up, deceased). Information on the current health care provider, the age at the first encounter with the specialized center, the age at diagnosis, and the specific diagnosis using Orphanet coding is also captured.

To further specify the diagnosis, additional data elements include availability and type of genetic diagnosis (eg, molecular or chromosomal). Details such as gene name(s), variant description according to nomenclature, allelic state, variant classification according to the American College of Medical Genetics and Genomics (ACMG) classification, and inheritance pattern were included as well.

### PROMs and Questionnaires (Level 1)

The dataset includes 15 data elements concerning PROMs and questionnaires. These elements consist of parent-reported outcome measures, such as the Intelligibility in Context Scale^13^ and the breathing scale of the Paediatric Sleep Questionnaire.^14^ In addition, quality-of-life assessments are captured using patient-reported outcome measures, such as the Paediatric Quality of Life Inventory.^15^ Several scales from the FACE-Q questionnaire^16^ and CLEFT-Q questionnaire^17^ are also included, covering the domains of facial function (speech, breathing, eyes, eating and drinking, and facial function), appearance (face, jaws, ears, and teeth), and health-related quality of life (psychological function, school function, and speech distress).

### Patient Characteristics (Level 2)

The dataset contains 28 data elements related to patient characteristics. These elements capture whether anomalies were detected prenatally, as well as the type of prenatal imaging and/or prenatal genetic tests used. In addition, the dataset includes information on consanguinity, maternal diseases during pregnancy, maternal medication use during pregnancy, maternal age at conception, and gestational age at birth. Furthermore, weight, height, and whether the patient follows mainstream or special education are registered. Congenital orodental anomalies are also collected, such as dental agenesis, supernumerary teeth, irregular tooth morphology, abnormalities in tooth eruption or position, and mandibular deformity (ie, Pruzansky classification^18^). Furthermore, craniofacial and extracranial anomalies are included, such as ocular, lacrimal, external ear, and middle ear anomalies (ie, Nagata classification,^19^ Altman/Cremers classification^20^), as well as limb, cardiac, vertebral, and trunk anomalies.

### Clinical Outcome Measures (Level 2)

The dataset contains 24 data elements related to clinical outcome measures. These elements capture occlusal and dental characteristics, such as overjet, overbite, maximal interincisal opening, occlusion characteristics (eg, open bite, cross-bite), and the Decayed, Missing, and Filled Teeth (DMFT) index.^21^ Vision-related data elements concern the degree of eyelid closure, visual acuity, refraction, and the presence of secondary ocular or adnexal anomalies. In addition, neonatal hearing screening results and subsequent air- and bone-conduction thresholds are collected.

Feeding-related measures are also part of the dataset, documenting occurrences such as choking, coughing, or nasal regurgitation during feeding, and a history of pneumonia, as well as the Functional Oral Intake Scale and the methylene blue dye test.^22,23^ Speech outcomes are captured using the perceptual ratings of velopharyngeal competence (VPC-rate) and percentage of consonants correct (PCC-A and/or PCC-R).^24,25^

### Imaging and Diagnostics (Level 2)

The dataset includes 31 data elements related to imaging and diagnostics. These elements capture the availability of orthopantomograms, lateral cephalograms, dental models, (cone-beam) CT scans, and temporal bone CT scans. In addition, the availability of photographic records is reported, such as 2D and 3D facial photography, intraoral photography, and photographs of the limbs. The dataset also registers endoscopic assessments of the airway, including the level(s) of airway obstruction. Similarly, diagnostic evaluations for swallowing, such as fibreoptic endoscopic evaluation of swallowing (FEES) and videofluoroscopic swallow studies (VFSS) are registered, including the result of the penetration-aspiration scale^26^ and the bolus residue scale.^27^ When polysomnographies are performed, the dataset also allows for detailed registration of the results (eg, Apnea-Hypopnea index, Nocturnal gas exchange). Finally, the specific genetic tests that were used for postnatal molecular diagnostic confirmation are included.

### Treatment Information (Level 2)

The dataset comprises 12 data elements related to treatment information, capturing (temporary) airway, and feeding interventions (eg, continuous positive airway pressure), as well as (non-)surgical hearing interventions and dental and orthodontic treatments. It also includes craniofacial and extracraniofacial surgical interventions, airway management during these surgeries, intubation methods, intubation difficulty (ie, Modified Cormack and Lehane Score^28^), and surgical complications. Finally, to quantify the treatment burden, both the operative duration and length of hospital stay for each surgery are also registered.

## DISCUSSION

The aim of this article was to describe the process of developing an internationally applicable and comprehensive dataset that can be used to monitor, evaluate, and compare the quality of care for FDS across Europe. By promoting standardized data collection across multiple centers, the dataset addresses the lack of uniform monitoring protocols, paving the way for collaborative research and filling evidence gaps on optimal diagnostic and treatment practices for these rare conditions. This initiative marks the first international registry for patients with FDS, offering significant potential for benchmarking, cross-center comparisons, and substantial improvements in patient care worldwide.

One of the dataset’s key strengths is its 2-level design, which is intended to improve accessibility and participation across centers with varying resources. While developing the cleft lip and palate dataset within ERN CRANIO, it was noted that clinicians across Europe often face high workloads, limiting the time available during outpatient clinics to create an environment where patients feel at ease and can openly discuss their concerns.^29^ The level 1 dataset, which consists exclusively of patient-reported outcome measures (PROMs), prioritizes outcomes that matter most to patients or that cannot be captured otherwise, such as emotional and psychosocial well-being. This way, the data collected for the level 1 registry is directly usable in the clinical setting, as essential information about the patient’s perspective is systematically gathered and ensures these topics are effectively communicated to the treating clinicians.

While the level 1 dataset enables benchmarking and intercenter comparisons, limitations in the use of PROMs must be acknowledged. First of all, PROMs alone do not perfectly evaluate the treatment provided, as they can be influenced by cultural differences, health literacy, fatigue bias, proxy response bias, and other factors.^30–32^ Thus, while the level 1 registry mainly focuses on the overall comparison of the quality of care from the patient’s perspective, the level 2 dataset is essential for more comprehensive benchmarking, as it incorporates detailed clinical data to provide a more elaborate understanding of the best care pathway for FDS. Furthermore, it is important to consider the burden of PROMs on patients and their families, given the large number of PROM scales in the dataset. However, an assessment of the workload among our patient representatives for all 12 Cleft-Q and Face-Q scales found that they could be completed in ∼10​​​​​ ​minutes in total, indicating that the overall burden for patients may be manageable. Nonetheless, it remains important to actively communicate the value and importance of these measures to maintain high response rates and ensure a positive experience for participants.

A significant strength of the development process was the inclusion of a multidisciplinary panel of experts and patient representatives. This approach ensured that diverse perspectives were considered and strengthened both the comprehensiveness and international applicability of the dataset. However, because of its extensive nature, some centers may need to make adjustments to fully incorporate it into their existing workflows, medical record systems, or hospital infrastructure. Besides, not all centers may be able to implement every data element due to constraints such as existing agreements for data collection or the absence of specific services like polysomnography or speech assessment.

Despite these considerations, even partial use of the dataset can still provide valuable insights and improve care for patients with FDS. Moreover, the endorsement of these data elements by a large panel of experts highlights their importance and can assist centers in establishing or expanding the necessary services over time (eg, polysomnography). The aim for every center is to at least complete the level 1 dataset, with the possibility of adding as many level 2 elements as resources allow. By periodically assessing the dataset’s use in practice, it can be refined to remain both practical and comprehensive, ultimately benefiting patients and their families, as well as clinicians.

A noteworthy observation is that none of the data elements were strongly discouraged during the Delphi voting process. This outcome likely reflects the selection process before voting, during which data elements that were deemed unreliable in the literature, such as the Epworth Sleepiness Scale and Brouillette score for obstructive sleep apnea syndrome screening,^33^ were excluded. Alternatively, the criteria used to categorize a data element as “strongly discouraged” during the Delphi process may have been too strict. This might unintentionally convey the impression that no data elements are discouraged for care quality assessment in FDS, which is not the case.

In conclusion, the newly developed FDS dataset marks a significant step toward achieving standardized data collection for these rare craniofacial conditions across Europe. By employing a 2-level design and drawing upon the expertise of a diverse panel of experts and patient representatives, the dataset remains accessible to a broad range of centers with varying resources. As the first international registry for FDS, it offers considerable potential for collaborative research, cross-center comparisons, and substantial improvements in patient care worldwide.

## Supplementary Material

SUPPLEMENTARY MATERIAL
