# Supplementary material for: The Development of a European Registry for Facial Dysostosis Syndromes: A Delphi-Guided Approach
Source: J Craniofac Surg. 2025 Jul 23;36(8):2712–6. doi: 10.1097/SCS.0000000000011695 (PMC12537031; doi:10.1097/SCS.0000000000011695)
Supplement: SUPPLEMENTARY MATERIAL [file scs-36-02712-s001.docx]

Supplemental Digital Table 1 – Characteristics of Expert panel and Delphi respondents

|  | **Expert panel (n=28)** | **Delphi Respondents (n=64)** |
| --- | --- | --- |
| Number of invitations sent | - | 149 |
| Response rate | - | 43.0% |
|  |  |  |
| **Profession** |  |  |
| Anaesthesiologist | 1 (3.6%) | 1 (1.6%) |
| Audiologist | 0 (0%) | 1 (1.6%) |
| Clinical geneticist | 3 (10.7%) | 5 (7.8%) |
| ENT specialist / Otolaryngologist | 2 (7.1%) | 7 (10.9%) |
| Maxillofacial surgeon | 3 (10.7%) | 11 (17.2%) |
| Nurse practitioner | 1 (3.6%) | 3 (4.3%) |
| Ophthalmologist | 2 (7.1%) | 2 (3.1%) |
| Orthodontist | 2 (7.1%) | 5 (7.8%) |
| Orthopaedic surgeon | 1 (3.6%) | 1 (1.6%) |
| Patient representative | 3 (10.7%) | 3 (4.7%) |
| Paediatric pulmonologist | 1 (3.6%) | 2 (3.1%) |
| Paediatrician | 1 (3.6%) | 2 (3.1%) |
| Plastic surgeon | 3 (10.7%) | 6 (9.4%) |
| Psychologist | 2 (7.1%) | 6 (9.4%) |
| Radiologist | 0 (0%) | 3 (4.7%) |
| Speech and Language Pathologist | 3 (10.7%) | 6 (9.4%) |
|  |  |  |
| **Hospital (Country code)** |  |  |
| Erasmus Medical Centre (NL) | 11 (39.3%) | 17 (26.6%) |
| Helsinki University Hospital (FIN) | 1 (3.6%) | 5 (7.8%) |
| Hôpital Universitaire Necker-Enfants Malades (F) | 4 (14.3%) | 7 (10.9%) |
| Hospital 12 de Octubre (E) | 1 (3.6%) | 5 (7.8%) |
| Hospital de Santa Maria (P) | 0 (0%) | 1 (1.6%) |
| Oslo University Hospital (N) | 1 (3.6%) | 2 (3.1%) |
| Sahlgrenska University Hospital (S) | 2 (7.1%) | 3 (4.7%) |
| San Gerardo Hospital (I) | 0 (0%) | 2 (3.1%) |
| San Paolo Hospital (I) | 0 (0%) | 1 (1.6%) |
| University Medical Centre Utrecht (NL) | 0 (0%) | 8 (12.5%) |
| Uppsala University Hospital (S) | 4 (14.3%) | 7 (10.9%) |
| Vall d'Hebron Hospital (E) | 1 (3.6%) | 3 (4.7%) |
|  |  |  |
| Patient organizations  (LAPOSA, Rare Diseases Croatia) | 3 (10.7%) | 3 (4.7%) |
|  |  |  |

Supplemental Digital Table 2 - Categorization of data elements, examples, and principal questions in the survey.

| **Category** | **Examples** | **Principle question** |
| --- | --- | --- |
| Patient characteristic | Height, gestational age, consanguinity, phenotypical anomalies | Should this element be registered to enable interpretation of outcome measures? /  Should this element be registered to allow for correction for case-mix in the future?" |
| Treatment information | Orthodontic treatment, feeding support, airway support |  |
| Clinical outcome measure | Occlusion class, visual acuity, pure tone average | Should this element be used to monitor, evaluate, and compare the quality of care for patients with FDS (inter)nationally? |
| PROMs and questionnaires | Face-Q scales, intelligibility in context score, paediatric quality of life inventory |  |
| Imaging and diagnostics | Facial photography, (CB)CT scan, orthopantomogram | Should the availability of this element be registered to monitor, evaluate, and compare the quality of care for patients with FDS (inter)nationally?" |
