# Supplementary material for: The Development of a European Registry for Facial Dysostosis Syndromes: A Delphi-Guided Approach
Source: J Craniofac Surg. 2025 Jul 23;36(8):2712–6. doi: 10.1097/SCS.0000000000011695 (PMC12537031; doi:10.1097/SCS.0000000000011695)
Supplement: SUPPLEMENTARY MATERIAL [file scs-36-02712-s002.docx]

**Supplemental Digital File 1 - Search Strings**

| **Database searched** | **Platform** | **Years of coverage** | **Records** | **Records after duplicates removed** |
| --- | --- | --- | --- | --- |
| Medline ALL | Ovid | 1946 - Present | 2370 | 2367 |
| Embase | Embase.com | 1971 - Present | 2703 | 752 |
| Web of Science Core Collection* | Web of Knowledge | 1975 - Present | 1378 | 329 |
| Cochrane Central Register of Controlled Trials | Wiley | 1992 - Present | 4 | 1 |
| CINAHL Plus | EBSCO | 1982 - Present | 183 | 32 |
| **Total** | | | **6638** | **3481** |

*Science Citation Index Expanded (1975-present) ; Social Sciences Citation Index (1975-present) ; Arts & Humanities Citation Index (1975-present) ; Conference Proceedings Citation Index- Science (1990-present) ; Conference Proceedings Citation Index- Social Science & Humanities (1990-present) ; Emerging Sources Citation Index (2005-present)

No other database limits were used than those specified in the search strategies

**Medline**(Mandibulofacial Dysostosis / OR (((mandibulofacial* OR facial* OR Nager OR acrofacial* OR Miller) ADJ3 (dysostos*)) OR ((Postaxial* OR Post-axial*) ADJ3 (acrofacial* OR acro-facial) ADJ3 dysostos*) OR (Treacher ADJ3 Collins) OR Franceschetti*).ab,ti,kw.) NOT (news OR congres* OR abstract* OR book* OR chapter* OR dissertation abstract*).pt. NOT (exp animals/ NOT humans/)

**Embase**('mandibulofacial dysostosis'/de OR 'Nager acrofacial dysostosis'/de OR (((mandibulofacial* OR facial* OR Nager OR acrofacial* OR Miller) NEAR/3 (dysostos*)) OR ((Postaxial* OR Post-axial*) NEAR/3 (acrofacial* OR acro-facial) NEAR/3 dysostos*) OR (Treacher NEAR/3 Collins) OR Franceschetti* ):Ab,ti) NOT ([conference abstract]/lim) NOT ([animals]/lim NOT [humans]/lim)

**Web of science**TS=(((((mandibulofacial* OR facial* OR Nager OR acrofacial* OR Miller) NEAR/2 (dysostos*)) OR ((Postaxial* OR Post-axial*) NEAR/2 (acrofacial* OR acro-facial) NEAR/2 dysostos*) OR (Treacher NEAR/2 Collins) OR Franceschetti* ))) NOT DT=(Meeting Abstract OR Meeting Summary) AND LA=(English)

**Cochrane**((((mandibulofacial* OR facial* OR Nager OR acrofacial* OR Miller) NEAR/3 (dysostos*)) OR ((Postaxial* OR Post-axial*) NEAR/3 (acrofacial* OR acro-facial) NEAR/3 dysostos*) OR (Treacher NEAR/3 Collins) OR Franceschetti* ):Ab,ti)

**CINAHL**(MH Mandibulofacial Dysostosis OR TI(((mandibulofacial* OR facial* OR Nager OR acrofacial* OR Miller) N2 (dysostos*)) OR ((Postaxial* OR Post-axial*) N2 (acrofacial* OR acro-facial) N2 dysostos*) OR (Treacher N2 Collins) OR Franceschetti*) OR AB(((mandibulofacial* OR facial* OR Nager OR acrofacial* OR Miller) N2 (dysostos*)) OR ((Postaxial* OR Post-axial*) N2 (acrofacial* OR acro-facial) N2 dysostos*) OR (Treacher N2 Collins) OR Franceschetti*)) NOT PT(news OR congres* OR abstract* OR book* OR chapter* OR dissertation abstract*) NOT (MH animals+ NOT MH humans+)
